# Supplementary material for: The Impact of Charlson Comorbidity Index on the Functional Capacity of COVID-19 Survivors: A Prospective Cohort Study with One-Year Follow-Up
Source: Int J Environ Res Public Health. 2022 Jun 18;19(12):7473. doi: 10.3390/ijerph19127473 (PMC9223623; doi:10.3390/ijerph19127473)
Supplement: Supplementary file 1 [file ijerph-19-07473-s001.zip › ijerph-1760041-supplementary.pdf]

Table S1. Association between Charlson Comorbidity Index and distance covered (meters) in the 6MWT test after Covid-19 hospitalization

n=41	R^2	β	95% CI
Crude model	0.1022	-18.7	-34.7 to -2.6
Adjusted model [a]	0.3286	-23.0	-39.1 to -6.8

[a] Model adjusted for a propensity score including sex, days of hospitalization, and 1-minute sit-to-stand test (age is included in the score of the Charlson Comorbidity Index).
